# Fuchs' Heterochromic Iridocyclitis in an Italian Tertiary Referral Centre: Epidemiology, Clinical Features, and Prognosis

**DOI:** 10.1155/2016/1458624

**Published:** 2016-10-03

**Authors:** Massimo Accorinti, Giovanni Spinucci, Maria Pia Pirraglia, Simone Bruschi, Francesca Romana Pesci, Ludovico Iannetti

**Affiliations:** Department of Ophthalmology, Sapienza University of Rome, Rome, Italy

## Abstract

*Purpose*. To study epidemiology, clinical findings and visual prognosis of patients with Fuchs' Heterochromic Iridocyclitis (FHI).* Methods*. A retrospective analysis was performed on 158 patients with FHI. Thirty-five patients were observed only once; the remaining 123 had a mean follow-up of 30.7 months (50 of them had a mean follow-up of 63.5 months) and in those we assessed complications, medical and surgical treatment, and long-term visual prognosis.* Results*. Average age at uveitis diagnosis was 27.2 years and 18.3% of patients were children. Blurred vision (54.5%) and floaters (40.5%) were the most frequent presenting symptoms. Small to medium-sized keratic precipitates (95.6%), iris atrophy (86.8%), and vitreous opacities (91.2%) were the most common signs; the prevalence of cataract and IOP increase was 63.5% and 20.1%, respectively, and their incidence was 0.1 and 0.06 eye/year. Significant risk factor for visual loss was IOP increase at presentation (*p* = 0.02). At final examination 98% of the eye had a visual acuity ≥ 0.6, and topical (*p* < 0.001) and systemic (*p* < 0.001) corticosteroids therapy were used less frequently than before referral.* Conclusions*. FHI has a good visual prognosis, despite the significant incidence of cataract and glaucoma. A correct and prompt diagnosis might avoid unnecessary therapies and provide excellent visual outcomes.

## 1. Introduction

Ernst Fuchs was the first in 1906 to report both the clinical and pathologic features of a consistent number of patients with a chronic low grade anterior chamber inflammation, heterochromia, and cataract [1]. This association was named after as Fuchs' Heterochromic Iridocyclitis (FHI). Nowadays FHI is one of the most common forms of anterior uveitis, accounting for up to 8% of endogenous uveitis seen in referral center [2–4]. Usually unilateral, it is characterized by the presence of diffusely distributed small, white, stellate, or rounded keratic precipitates, low grade inflammation in anterior chamber, absence of posterior synechiae, diffuse iris stroma atrophy with or without heterochromia, and variable vitreous inflammation [1, 4–11]. It is not associated with systemic diseases and it is not or poorly responding to corticosteroid therapy. FHI affects both sexes equally, and the prognosis is usually good [12]. The onset is between 29 and 44 years of age [4, 7, 9–11, 13–17]. Iris atrophy and heterochromia are its most peculiar findings; although not pathognomonic, they are due to atrophy and depigmentation of all iris layers (anterior border, stroma, and pigmented epithelium) [3]. Usually the lighter eye is affected, although bilateral cases have been described as well as inverse heterochromia. The natural history of the disease is characterized by a slow progression over time, without substantial reduction of visual acuity until the development of significant vitreous opacities or cataract. A late or wrong diagnosis may lead to severe ocular complications, mainly related to a useless long-term corticosteroid therapy. The most frequent complications of FHI are cataract (70% of patients) and glaucoma (25%) [6, 7]. Glaucoma is often difficult to manage with medical therapy and may lead to a surgical intervention not always associated with good results. The visual prognosis of patients who undergo cataract surgery with intraocular lens implantation is usually good, with complete visual function recovery [18–20].

The purpose of the present study was to evaluate the clinical and epidemiological findings, the prevalence and incidence of complications, and the long-term visual prognosis of Italian patients with FHI.

## 2. Patients and Methods

A retrospective analysis of the clinical charts of patients referred to the Ocular Immunovirology Service of Sapienza University of Rome, Rome, Italy, from January 2003 to December 2012 was performed. The study was undertaken in accordance with the tenets of the declaration of Helsinki.

Inclusion criteria was a diagnosis of FHI, which has been made upon several of the following clinical features: (i) small to medium-sized keratic precipitates involving the whole endothelial surface; (ii) a chronic inflammation in anterior chamber, usually ≤2+ according to SUN criteria [21]; (iii) diffuse iris stromal atrophy with or without heterochromia; (iv) lack of posterior synechiae unless there was a history of ocular surgery; (v) absence of snowbanks or choroidal/retinal infiltrates despite the presence of vitreous cells.

One-hundred and fifty-eight patients, 80 females (50.6%) and 78 males (49.4%), fulfilled the criteria and were included in the study. One patient had a bilateral involvement.

After recording a detailed ocular and medical history, each patient underwent a full ophthalmological examination including best-corrected visual acuity (BCVA), slit-lamp biomicroscopy, bilateral indirect ophthalmoscopy and biomicroscopy of the fundus, and Goldmann applanation tonometry. In some unclear cases, in order to exclude other forms of uveitis, we performed additional laboratory examinations, including a complete blood cell count, erythrocyte sedimentation rate, tuberculin skin tests or quantiFERON-TB Gold, syphilis serology, serum biochemical analysis, chest X-ray, and angiotensin converting enzyme determination. Toxoplasma serology was done only in patients with chorioretinal scars.

For all the patients, we have recorded the following: age at uveitis diagnosis, age at FHI diagnosis, age at presentation at referral center, unilateral or bilateral involvement, clinical features at presentation, clinical course, forms of treatment, ocular complications at our first examination or occurring during the follow-up (when available), and visual prognosis.

Thirty-five patients were observed only once, while 123 subjects (124 eyes) were followed up for more than 3 months (mean follow-up: 30.74 ± 26.9 months; range: 3–119 months). Ocular complications and the need for medical and surgical treatment were evaluated on those last patients. A further evaluation to assess the long-term visual prognosis was done on 50 patients who had a follow-up longer than 36 months (mean: 63.47 ± 20.38 months; range: 36–119 months).

The statistical analysis was performed using the Student's *t*-test and the *χ* ^2^ test. *p* values ≤ 0.05 were considered statistically significant. Kaplan-Meier curves were used to compare eye complication rates. All the analyses were performed using SPSS 19.0 statistical software (LEAD Technologies).

## 3. Results

The epidemiologic findings of 158 patients with FHI are reported in Table 1.

The average age at uveitis diagnosis was 27.19 ± 10.61 years (range 7–61 years), while that of FHI was 29.22 ± 11.31 years (range: 8–61 years). The average age at our first examination was 32.69 ± 10.97 years (range: 8–64 years). The mean interval from uveitis diagnosis to examination in our referral center was 2.12 ± 1.7 years. There was no significant difference between the average age at uveitis onset between males (27.74 ± 10.54 years, range: 7–61 years) and females (26.66 ± 10.73 years, range: 8–50 years) (*t* = 0.64; *p* = 0.52).

In 29 patients (18.35%) FHI was diagnosed in children (before 16 years of age) and in 1 of those before 7 (0.6% of all the patients, 3.4% of the pediatric ones). FHI was diagnosed after 60 years of age in one patient only, during a routine eye examination.

Fifty-five patients (34.8%) had a positive history of rubella, while seventy-three patients (46.2%) had no history of rubella and were not investigated serologically. Thirty patients (18.98%) had no history of rubella and were serologically tested: all (100%) were positive for rubella IgG antibody.

Blurred vision and floaters were the most frequent presenting symptoms (86 patients, 54.5%, and 64 patients, 40.5%, resp.). Other symptoms recalled by the patients included hyperemia (25 patients, 15.8% of the cases), photophobia (24 patients, 15.1%), and pain (21 patients, 13.2%). Thirty-six patients were asymptomatic (23%), and the diagnosis was made during a routine eye examination.

The clinical findings of patients with FHI at our first observation are reported in Table 2.

One hundred and thirty-six eyes (86%) showed a BCVA ≥ 0.6, 16 (10.1%) between 0.2 and 0.5, and 6 eyes (3.8%) ≤ 0.1. Glaucoma was the leading cause of a severe visual loss in 50% of those cases (3 eyes, 1.88% of the eyes), followed by cataract, severe vitreous opacities, and corneal scar in 1 eye each (0.6%).

The most common clinical findings were small to medium-sized keratic precipitates (95.6% of the eyes). According to SUN criteria [21] no to moderate inflammation in anterior chamber (cells from 0 to ≤2+) was observed in 158 eyes (99.37%), and 1 eye only presented 3+ cells (0.6%). Sixty eyes (37.7%) presented iris nodules: Koeppe nodules in 48 cases (30.2%), Busacca in 2 (1.2%), and both types in 10 eyes (6.3%). Iris stroma atrophy was found in 138 eyes (86.8%), but only 61 (38.3%) presented heterochromia. In one patient an inverse heterochromia was found. Anisocoria was observed in 4 eyes (2.4%) and no patient had posterior synechiae. In 32 eyes (20.12%) we found an increased intraocular pressure (IOP): 27 have been already diagnosed and treated, while 5 were unaware of the condition (3.78% of all the patients with a normal IOP before our observation).

Fourteen eyes (8.8%) had undergone cataract surgery with intraocular lens (IOL) implantation elsewhere. Among the remaining 144 patients (145 eyes), 83 eyes presented a posterior subcapsular cataract (57.24%) and 4 eyes (2.75%) a white cataract. The prevalence of cataract was 63.5%. Vitreous opacities were found in 145 eyes (91.2%), where in 72 eyes (45.28%) they were ≥ 2+. Optic disc changes were found in 27 eyes (17%): 15 eyes (9.4%) presented a cup/disk ratio > 0.6, 7 (4.4%) a pale optic disc, 2 (1.2%) both of these changes, and 3 (1.9%) an optic disc hyperemia.

Chorioretinal scars were found in 8 eyes (5%). Serum antitoxoplasma IgG antibody was positive in 2 of these patients (25% of those with retinal scars), but none of the patients had a history of symptomatic ocular or systemic toxoplasmosis. Epiretinal membranes were found in 3 eyes (1.9%), retinal tear in 1 eye (0.6%), and a retinal hole in another one (0.6%), respectively.

Four patients with FHI (2.5%) presented a systemic disease prior to uveitis onset that might confuse the diagnosis, because of their possible association with uveitis: 2 (1.25%) were affected by psoriasis, 1 (0.6%) by celiac disease, and 1 by acute rheumatic disease. Two other patients (1.25%) developed a systemic disease potentially associated with uveitis during follow-up: one, serologically positive for rubella, developed an optic neuritis in FHI unaffected (and completely normal) eye, 19 years after FHI diagnosis, and was diagnosed as having multiple sclerosis on a nuclear magnetic resonance results and neurologic consultation; another patient developed Hodgkin's lymphoma 2 years after FHI onset.

Among 119 eyes which were followed up for a mean period of 30.74 ± 26.92 months, 29 (24.36%) which at baseline have had no cataract or an initial subcapsular cataract without significant reduction of visual acuity presented a BCVA reduction < 5/10 because of cataract onset/progression. The incidence of cataract was 0.1 eye/year. IOP increase developed in 15 of 91 eyes (16.48%), with an incidence of 0.06 eye/year. Epiretinal membranes were quite rare (2 eyes; incidence: 0.006 eye/year).

**Figure 1:**
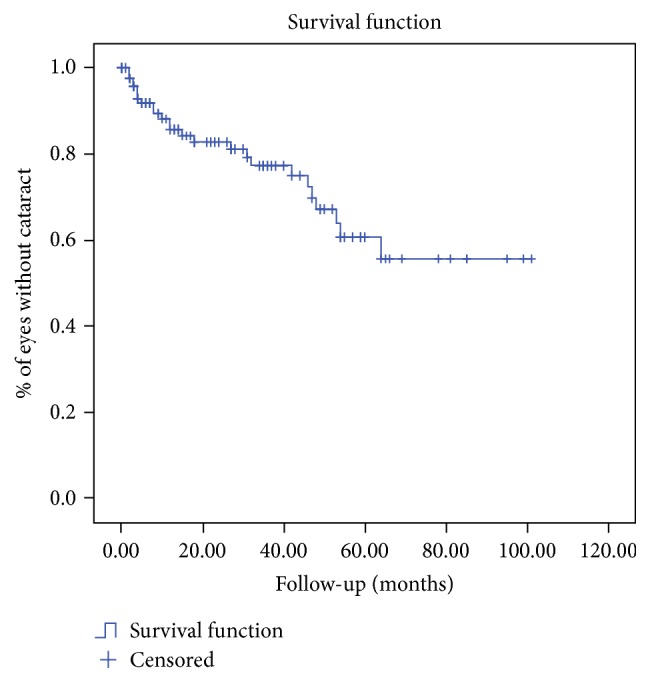
Kaplan-Meier method for assessing the risk of developing cataract in patients with Fuchs' Heterochromic Iridocyclitis. The Kaplan-Meier curve shows the percentage of eyes developing no cataract.

**Figure 2:**
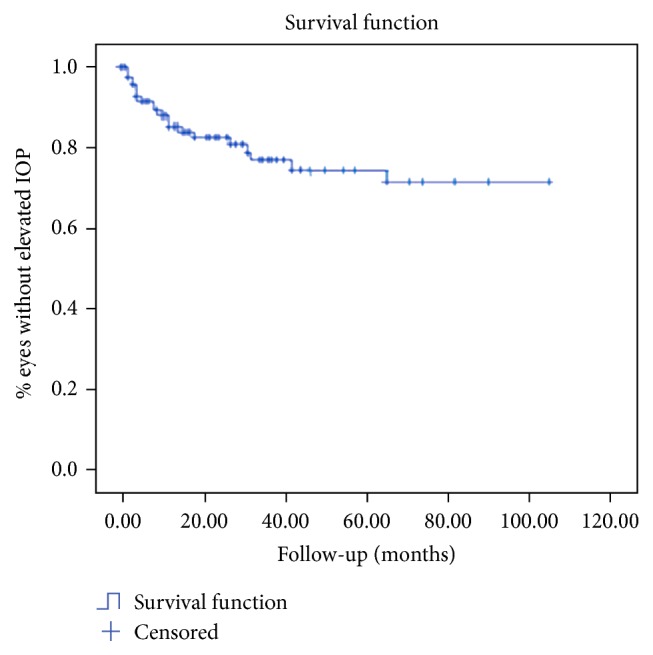
Kaplan-Meier method for assessing the risk of developing elevated IOP in patients with Fuchs' Heterochromic Iridocyclitis. The Kaplan-Meier curve shows the percentage of eyes developing no IOP increase.

Figures 1 and 2 show the Kaplan-Meier curve for assessing the risk of developing cataract and increased IOP.

The overall risk for a visual acuity reduction ≤ 0.4 was 0.001 eye/year.

Risk factors for visual acuity reduction are reported in Table 3.

The only significant risk for a visual acuity reduction ≤ 4/10 was an IOP increase at presentation (*p* = 0.02).

During our follow-up, 54 eyes (43.5%) had no therapy. Twenty-nine eyes (23.38%) were given a course of systemic corticosteroid therapy: 12 eyes (9.75%) to try to lower vitreous opacities and 17 (13.8%) to prevent and control postoperative (cataract and glaucoma) inflammation. Sixty-five eyes (52.4%) received topical corticosteroid therapy and 40 eyes (32.25%) mydriatics, mostly immediately after a surgical procedure (25 cases of cataract, 11 cases of glaucoma). Forty-seven eyes (37.9%) received IOP lowering drops.

Comparing the therapeutic regimen given before and during our follow-up, we have observed a significant decrease in the need for medications: topical steroids from 82.2% to 52.4% of the eyes (*χ* ^2^ = 23.76; *p* < 0.001) and systemic steroids from 58.8% to 23.38% (*χ* ^2^ = 30.79; *p* < 0.001). Eight patients (6.5%) had been previously subjected to immunosuppressive therapy elsewhere. These drugs were discontinued in all the cases during our follow-up (*χ* ^2^ = 6.33; *p* = 0.012).

In twenty-five patients (25 eyes) phacoemulsification and in the bag IOL implantation was performed by us (17.4% of the phakic patients at our first examination). BCVA at the end of follow-up was ≥ 0.8 in all the cases (mean 0.88 ± 0.04). Capsulotomy was performed in 13 patients (33.33% of all the patients who have undergone cataract surgery): in 7 out of 25 eyes operated by us (28%) and in 6 out of 14 eyes (42.85%) operated elsewhere (*χ* ^2^ = 0.34, *p* = 0.55).

Trabeculectomy was performed in 14 eyes (8.8% of all the patients; 33.3% of those with IOP increase), in 11 cases (6.9%) by us. The incidence of trabeculectomy was therefore 0.02 eye/year. The mean BCVA at the end of follow-up of eyes undergoing trabeculectomy was 0.61 ± 0.42, in 3 eyes less than 0.5 and in 2 less than 0.1. Both of these presented a very low preoperative visual acuity (light perceptions and 0.1, resp.).

In 50 patients (50 eyes), followed up for an average period of 63.47 ± 20.38 months (range: 36–119 months), we have observed a BCVA at baseline ≥ 0.6 in 45 eyes (90%) and ≤ 0.1 in 3 eyes (6%). At the end of follow-up one eye (2%) presented a BCVA ≤ 0.1, because of a corneal scar already present at the first examination, while all the others (98%) had a BCVA ≥ 0.6. At the final examination 2 eyes (4%) presented, compared to baseline, a BCVA reduction of 0.3 (cataract) and five (10%) presented an increase of BCVA of 0.3: in three cases after cataract surgery, in one after trabeculectomy, and in 1 because of vitreous opacities reduction after anti-inflammatory therapy.

## 4. Discussion 

FHI is a quite common type of uveitis and its diagnosis relies of clinical findings [4–12]. This is why diagnosis is often delayed, because general ophthalmologist might miss to analyze properly the specific clinical features such as small or medium-sized keratic precipitates distributed on the whole endothelial surface, iris atrophy or heterochromia, low or moderate chronic inflammation in anterior chamber, absence of synechiae, and variable vitreous involvement.

In our series the diagnosis of FHI was made on average 2 years after symptoms onset. Norrsell and Sjödell reported a period of 3 years between the onset of the first ocular symptoms and the diagnosis, while Fearnley and Rosenthal reported a delay of 6.7 years [9, 10]. This finding could be easily explained by the absence of heterochromia, which has been considered in the past the hallmark sign of FHI. It is of note that only 38% of patients had heterochromia at our first observation, performed on average 5 years after symptoms onset. Tugal-Tutkun et al. described heterochromia in 39% of cases while older studies reported higher prevalence (70–75%) [4, 9–11]. The lower prevalence of heterocromia found in tertiary referral center for uveitis might be due to the possibility that only the most difficult-to-diagnose cases are sent for referral, while the true prevalence of heterochromia in general FHI population might be higher. Iris atrophy is definitely more common than heterocromia in Italy (87% of the eyes). Although two series from Brazil and Spain reported a low frequency of iris atrophy (18% and 14.8%) [8, 11], this finding can be found in 48 to 100% of patients from different countries [4, 6, 8–11, 14, 16]. Therefore iris atrophy should be considered a more appropriate clinical feature to be associated with FHI.

The mean age of uveitis diagnosis was found to be 27 years, lower than reported by other authors (29.5 to 44.5 years) [4, 6, 8–11, 16], with no significant differences between genders, as already described [7, 9, 10]. Our study has confirmed that FHI can also appear before 16 years of age in almost 20% of the patients. Tappeiner reported on FHI onset in childhood in 8% of German patients, 26% of whom were before 7 years of age [22]: this last finding seems to be more rare in Italy (0.63% of the patients; 3.4% of the pediatric ones).

FHI etiology remains unknown. Several theories have been proposed and, among the possible causes, some viral (rubella, cytomegalovirus) and parasitic (*Toxoplasma gondii*) agents were suggested. Quentin and Reiber detected the presence of anti-rubella antibodies, using the Goldmann-Witmer index, in 100% of FHI patients [23]. Similar results were confirmed by other authors [24–26]. Birnbaum et al. demonstrated a significant decline in the prevalence of FHI after the introduction of a rubella vaccination program [27]. In our series 34.8% of patients had a positive history of rubella infection and an additional 19%, who did not remember any rubella infection, tested positive serologically. Cytomegalovirus DNA has been also found by PCR in aqueous humor of patients with a clinical diagnosis of FHI, both in Asia [28] and in Europe [29]. The association with* Toxoplasma gondii* was also described, but it is controversial [5, 30, 31]. The presence of chorioretinal scars in patients with FHI has been reported for decades, from 0% in a large group of Chinese patients to 28% in Brazil, a country where toxoplasmosis is endemic [13]. Only 25% of our patients with FHI and chorioretinal scars tested positive serologically for IgG antitoxoplasma antibody: it seems therefore reasonable to agree with Kreps et al. who have reported that the findings of the last decade show that rubella virus is the major—but likely not sole—etiologic agent in FHI [32].

Blurred vision and floaters were the most frequent presenting symptoms (54.5% and 40.5% resp., in our patients), similar to Turkish population [4], but significantly lower than in Sweden (71%) and England (83.9%) [9, 10]. Although the classic description of FHI does not include a ciliary reaction, redness photophobia and pain have been recorded at the uveitis diagnosis in almost 15% of our cases.

Twenty-three percent of Italian patients with FHI was completely asymptomatic, and the diagnosis was made during a routine eye examination.

A bilateral involvement was found in one of our patients (0.6%) only, while this frequency varies in the literature from 0% to 21% [10]. Norrsell and Sjödell have described a worse prognosis in bilateral cases, with more frequent cataract extraction and pars plana vitrectomy [10].

Small to medium-sized keratic precipitates are typical of FHI [4–11] and have been found in 96% of our patients. At their first observation they were either equally diffuse on the whole endothelium (76.3%) or mainly located in the central and inferior part (23.7%) A recent study has shown a close association between endothelial precipitates and CMV-DNA in the aqueous humor, suggesting that the endothelial cells may be the target of a viral infection [33]. The presence of iris nodules (37.7% in our patients, mostly Koeppe's one: 30.2%), with no posterior synechiae and with a low to moderate inflammation in anterior chamber (cells ≤2+ in 99.4% of the our cases), is similar to the clinical findings reported in literature [4] and should strongly suggests the diagnosis of FHI. Vitreous inflammation frequency has been reported from 14.8 to 92.6% in different series [4, 6, 8–11, 14, 16, 34]. It was found by us in 91.2% of patients, 45% of whom showed vitreous opacities >2+. Again this frequency can be attributed to each author's affiliation, working with general population or in referral center for uveitis. Nevertheless it is important to stress that vitreous opacities, even ≥2+, can be easily found in FHI, especially in long-standing cases, and might be a confounding finding leading to a wrong diagnosis of intermediate or posterior uveitis, especially when the iris atrophy and heterochromia are not clearly detectable. In such cases the presence of a normal foveal reflex and the absence of choroidal, retinal, or vascular lesions, of snowballs in the far periphery, are elements that should lead to diagnose FHI. Epiretinal membranes are rare in FHI.

Cataract becomes mature on average 8 years after first subcapsular opacities have been detected but can also develop very rapidly. In our study, a posterior subcapsular cataract was observed in 57.2% of phakic eyes at the first examination, while the overall prevalence was 63.5%, similar to Turkish patients (69%) [4]. The highest frequencies of cataract in FHI were described by Liesegang (90%) and Tabbut et al. (75%) [14, 15]. Visual prognosis in FHI after cataract surgery is better than that reported in other forms of uveitis [35, 36]. Fourteen patients before our observation and 25 patients during our follow-up (total: 39 patients, 24.7% of all the cases) underwent cataract surgery with IOL implantation, confirming previous results with a final visual acuity ≥ 0.8 in all cases. The clinical characteristics of FHI such as low anterior chamber inflammation, absence of posterior synechiae and macular edema, and benign long-term prognosis make this uveitis an ideal candidate for IOL implantation. In contrast, chronic inflammation in FHI might justify the high prevalence of posterior capsule opacification. In 33.3% of patients who underwent cataract surgery, we observed a posterior capsule opacification requiring Nd: YAG laser capsulotomy. This percentage was higher than that found in senile cataract (20 to 40%) [37, 38], but it seems possible to lower the opacification rate with a meticulous removal of all the cortex and probably a more aggressive treatment tailored on a precise knowledge of the preoperatively inflammatory status [36]. In fact we have observed a lower prevalence of posterior capsule opacification after cataract extraction and in the bag IOL implantation comparing our patients (28%) to those operated elsewhere (42.8%).

The prevalence of an increased IOP and/or secondary glaucoma in patients with FHI is variable, with literature data ranging between 6.3% and 59% of cases [4, 10]. The onset of IOP increase is difficult to determine because it might remain unrecognized for a long period of time. In our patients the prevalence of increased IOP was 20.1% and the incidence, during a mean follow-up of 30 months, was 0.06 eye/year. So at the end of our observation, the prevalence of IOP alterations was 33.87%. La Hey et al. and Jones reported in FHI a failure of medical therapy in 37% and in 73% of the cases, respectively [12, 16]. According to Fearnley and Rosenthal, glaucoma surgical approach in FHI patients is useful in 47% of eyes, while Liesegang performed glaucoma filtering surgery in 66% of their patients [9, 14]. In our study, 11 eyes during follow-up and 3 before our first observation underwent trabeculectomy (8.8% of all the patients, 33.3% of the patients with IOP elevation) with an incidence of 0.03 eye/year. In these patients the mean postoperative visual acuity was 0.61 ± 0.42, while the only 2 patients who had a final visual acuity equal to or less than 0.1 had a preoperative visual acuity of light perceptions and 0.1.

The visual prognosis of FHI is usually considered good. Our study has demonstrated that the incidence of visual decrease ≤ 0.4 is very low in medium term follow-up (0.001 per eye/year). Al-Mansour et al. reported a worsening of visual acuity only in 10% of the eyes compared to baseline, while most of the eyes had a visual acuity unchanged or improved [34]. In our patients the risk of developing a reduction in visual acuity is significantly related to the presence of an increased IOP or glaucoma at the first observation (odds ratio 10.11, *p* = 0.02), similar to Al-Mansour et al.'s experience [34].

In follow-up longer than 5 years we have observed a final visual acuity > 0.6 in 98% of the patients, with one patient only presenting a poor visual acuity related to a previous corneal scar. FHI is often misdiagnosed and patients are treated with topical, systemic, and peribulbar steroids. Peribulbar or topical steroid treatment has a minimal effect on this inflammation and it might promote the onset of posterior subcapsular opacity and elevated IOP. The referral of patients in tertiary eye care center can significantly lower the amount of corticosteroids therapy (*p* < 0.001 for both topical and systemic route of administration) and of immunosuppressive drugs (*p* = 0.012). These might potentially reduce the incidence of cataract and glaucoma, which are typical side effects of such a therapy, and, more importantly, of immunosuppressives-related systemic side-effects.

## 5. Conclusions

In conclusion in Italian patients the average age at presentation is lower than in other countries, and 18% of patients are aged 16 or less. Therefore in children with unilateral, chronic anterior uveitis, it is essential to exclude FHI to avoid the incidence of therapy-related side-effects. The clinical findings of FHI do not differ from those reported in other countries, but particular attention should be paid for iris atrophy and vitreous opacities, which resulted in being more frequently encountered than heterochromia. Our study confirmed that FHI has a good long-term visual prognosis, despite the significant incidence and prevalence of cataract and glaucoma; the only significant risk factor for visual loss is IOP increase. During cataract management it is important to remove all cortical residuals and follow adequately the patient in the postoperative period to reduce the incidence of posterior capsule opacification. A correct and prompt diagnosis allows to avoid unnecessary and potentially dangerous therapy. A proper uveitis, and its complications, management can lead to a final excellent visual outcome.

## Figures and Tables

**Table 1:** Epidemiologic findings of 158 Italian patients with Fuchs' Heterocromic Iridocyclitis (FHI).

Average age at uveitis diagnosis (years)	27.19 ± 10.61
Males	27.74 ± 10.54
Females	26.66 ± 10.73

Average age at FHI diagnosis (years)	29.22 ± 11.31

Average age at our first examination (years)	32.69 ± 10.97

Pediatric FHI (uveitis diagnosis < 16 years)	29 (18.35%)

Senile FHI (uveitis diagnosis > 60 years)	1 (0,6%)

History of rubella infection	55 (34.8%)

**Table 2:** Clinical findings of 158 Italian patients (159 eyes) with Fuchs' Heterochromic Iridocyclitis at their first examination in a referral center.

Clinical findings	Number of eyes	%
BCVA ≥ 0.6	137	86

BCVA ≤ 0.1	6	3.8

Keratic precipitates	152	95.6
Small size	67	42.1
Medium size	27	17
Small + medium size	58	36.5
Diffuse	116	76.3
Mainly central and inferior	36	23.7

Cells in anterior chamber	122	76.7
0 to ≤2+	158	99.37
≥3+	1	0.6

Iris nodules	60	37.7
Koeppe	48	30.2
Busacca	2	1.2
Koeppe + Busacca	10	6.3

Iris atrophy	138	86.8
Heterochromia	61	38.3
Inverse heterochromia	1	0.6

Anisocoria	4	2.5

IOP increase or therapy for IOP	32	20.1

Cataract	101	63.5
Posterior subcapsular	83	57.2^*∗*^
White	4	2.75^*∗*^
Previous cataract surgery	14	8.8

Vitreous opacities	145	91.2
≤1+	73	45.9
≥2+	72	45.3

Optic disc changes	27	17
Increased cup/disk ratio	15	9.4
Pale optic disk	7	4.4
Both	2	1.2
Hyperemia	3	1.9

Chorioretinal scars	8	5

Epiretinal membrane	3	1.9

Retinal tears	1	0.6

Retinal hole	1	0.6

^*∗*^On 145 phakic eyes.

**Table 3:** Risk factors for visual acuity ≤ 0.4 during follow-up in eyes with visual acuity ≥ 0.5 at our first examination.

	Odds ratio (95% confidence interval)	*p*
Age < 30 years at uveitis diagnosis	0.25 (0.02–2.81)	0.22
Interval > 24 months between uveitis diagnosis and our first examination	0.56 (0.05–6.4)	0.64
Males versus females	2.38 (0.21–27.05)	0.47
Iris nodules	0.9 (0.08–10.26)	0.93
Heterochromia	0.95 (0.08–10.66)	0.95
Vitreous opacities > 2+	2.26 (0.2–25.62)	0.49
Increased IOP	10.11 (0.87–117.15)	0.02
